# Zika Virus and the Risk for Renter Households

**DOI:** 10.3390/diseases6020037

**Published:** 2018-05-15

**Authors:** Amanda Scarbrough, Heranga Rathnasekara, Melinda Holt, Jack Hill, Ram Kafle

**Affiliations:** 1 Department of Population Health, Sam Houston State University, Huntsville, TX 77340, USA; 2 Department of Mathematics and Statistics, Sam Houston State University, Huntsville, TX 77340, USA; hkr009@shsu.edu (H.R.); MXM014@SHSU.EDU (M.H.); rck020@shsu.edu (R.K.); 3 Office of Research and Sponsored Programs, Sam Houston State University, Huntsville, TX 77340, USA; jmh050@SHSU.EDU

**Keywords:** social determinants of health, Zika virus, renters

## Abstract

Recent research on family income indicates that a lack of economic stability can affect healthy housing. Those with limited resources experience higher rates of inadequate and unstable housing many times forcing them to live in undesirable communities in which there can be several community-level health-related issues. One community-level health-related factor of concern has been the reemergence of Zika virus. Some research has indicated that a higher risk of catching Zika virus may exist in neighborhoods and areas with unhealthy housing. Therefore, this study sought to explore the existence of a relationship between rental housing and the Zika virus. Our findings indicated a significant correlation existed between renter occupied household units and the presence of Zika virus. This finding is notable as it indicates that renters have a higher chance of contracting Zika virus than non-renters. Future research should further examine the demographic and housing situation in other communities reporting cases of the Zika virus.

## 1. Introduction

Social determinants of health have a significant impact on the heath of individuals [1,2]. The environments in which people are born, live, learn, work, play, worship, and age affect a wide range of health, functioning, and quality-of-life outcomes and risks [1]. According to Healthy People 2020 five key determinants of health exist: education, social and community context, health and health care, neighborhood, and built environment and economic stability [3].

Recent research on economic stability indicates that, generally, higher income is linked to better health. The greater a person’s income is, the lower that person’s likelihood of disease and premature death [4]. For example, poor adults are almost five times as likely to report being in fair or poor health as adults with family incomes at or above 400 percent of the federal poverty level [5]. Low-income American adults have higher rates of heart disease, diabetes, stroke, and other chronic disorders than wealthier Americans [6].

In addition to negatively influencing physical health, a lack of economic stability can affect healthy housing. A healthy home is sited, designed, built, renovated, and maintained to support health [7]. Those with limited resources experience higher rates of inadequate and unstable housing. A lack of economic stability can preclude individuals from being able to afford to live in healthier, more desirable areas leading them to struggle with the challenges related to a variety of community-level health-related factors [6].

One community-level health-related factor of concern has been the reemergence of Zika virus. Zika is a virus spread through the bite of the as *Ae. aegypti* and *Ae. albopictus* mosquito, sex and from a pregnant woman to the fetus and can cause fever, rash, headache, joint pain, red eyes, muscle pain and microcephaly in unborn children [8].

Some research has indicated that a higher risk of catching Zika virus may exist in neighborhoods and areas with unhealthy housing [9]. Therefore, this study sought to explore the existence of a relationship between housing and the Zika virus. This analysis was exempt from Institutional Review Board (IRB) approval because it utilized existing data that were publically available and in which the subjects could not be identified.

## 2. Methods

### 2.1. Sample

**Figure 1:**
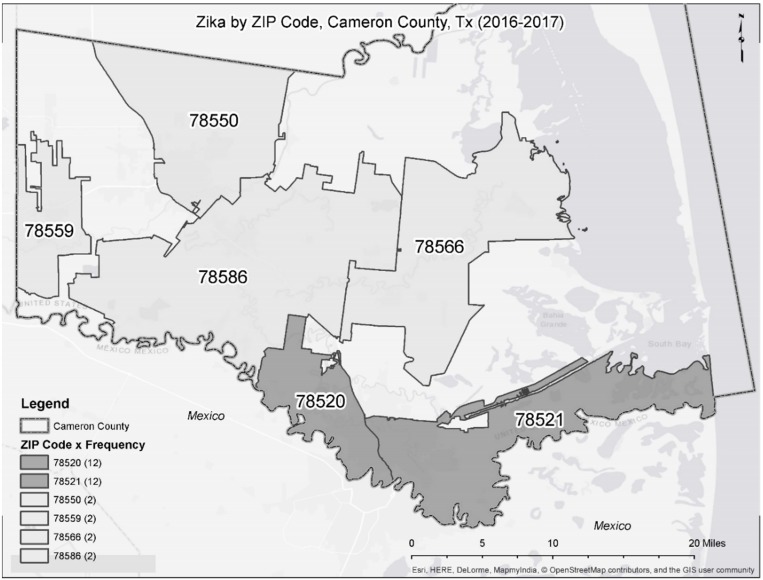
Zika by ZIP Code, Cameron County, Tx (2016–2017).

Cameron County, Texas sits on the border between the US and Mexico (see Figure 1). As of the census of 2010, there were 406,220 people, 119,631 households, and 96,579 families residing in the county. The racial makeup of the county was 87.0% White, 0.5% Black or African American, 0.4% Native American, 0.7% Asian, 0.03% Pacific Islander, 9.8% from other races, and 1.5% from two or more races. 88.1% of the population were Hispanic or Latino of any race. There were 119,631 households out of which 50.3% had children under the age of 18 living with them. The average household size was 3.36 and the average family size was 3.80. The population was 33.0% under the age of 18, 9.7% from 18 to 24, 25.6% from 25 to 44, 20.6% from 45 to 64, and 11.10% who were 65 years of age or older. The median income for a household in the county was $31,264, and the median income for a family was $33,770. Approximately, 30.0% of families and 34.7% of the population were below the poverty line [10].

According to the Cameron County Department of Health and Human Services, there were 5227 Zika virus cases in United States and 329 Zika virus cases in Texas including 32 Zika virus cases in Cameron County in 2016 and 2017 (until 1 April 2017). These 32 Zika virus cases were reported from six of the 22 Zip codes. Accordingly, the 16 Zip codes in Cameron County including these 6 Zip Codes in which Zika virus occurred was selected as the sample (The information was available only for 16 Zip Codes). Table 1 lists the Zip code name with the corresponding count of Zika virus cases (see Table 1).

### 2.2. Data Collection

Data regarding Zika virus occurrences in Cameron County were acquired from Cameron County Department of Health and Human Services. Demographic and socioeconomic information was collected using data from the Environmental Systems Research Institute (ESRI), Community Analyst and the US Census. Eight demographic and socioeconomic variables were gathered as the independent variables.

### 2.3. Dependent Variable

The number of Zika virus cases in Cameron County was gathered to identify the possibilities to either prevent or reduce to the spread of Zika virus across the area.

### 2.4. Independent Variables

The independent variables were: the Hispanic Population Percentage, (Hispanic refers to a person of Cuban, Mexican, Puerto Rican, South or Central American), the Vulnerable People’s Age Percentage (The percentage of people who are less than age 9 and more than age 65), Renter Occupied Housing Units Percentage, (Renter Occupied refers to a housing unit wherein the dweller pays to live in a housing unit owned by someone else), Vacant Housing Units Percentage, Median Disposable Income per Household, (Median Disposable Income refers to the amount of funds a household has after spending for bills), Average Household Size, Average Money Available per Person per Household and Higher Education Percentage (The percentage of the people who has at least a bachelor’s degree, graduate/professional degree or an associate degree) in each selected Zip code in Cameron County.

## 3. Results

Data were analyzed using Version 23 of the IBM Statistical Package for the Social Sciences (SPSS). Pearson Correlation was used to test for bivariate correlation between the Zika virus count and all the other eight independent variables. Then an independent t-test was performed for the variables which were selected as statistically significant from the bivariate correlation. Moreover, the Levene’s Test for equal variance and normality test were performed in order to test for the assumptions in independent T test. Finally, the Mann Whitney’s test was conducted to identify which independent variable(s) has a relationship with the Zika virus case occurrence.

In the sample, on average 81.6% of the population were Hispanic and 15.25% were in vulnerable age. With respect to housing, 20.66% of the Housing Units were Renter Occupied and 19.96% were Vacant Housing Units. Median Disposable Income per Household had a roughly even spread with 42.73% reporting an income of less than $30,000. Average Household Size was 3.3 and Average Money Available per Person per Household was $10,832. With respect to education, 24% of the people who were more than 18 years of age had at least a bachelor’s degree, graduate/professional degree or an associate degree.

There was a correlation between Renter Occupied Household Units and Vacant Housing Units (r = −0.498, *p* = 0.049). None of the other independent variables were correlated with Renter Occupied Household Units. All the other independent variables were correlated with Vacant Housing Units; Vacant Housing Units and Hispanic Population (r = −0.952, *p* = 0.000), Vacancy Housing Units and Vulnerable People’s Age (r = 0.753, *p* = 0.001), Vacant Housing Units and Median Disposable Income per Household (r = 0.748, *p* = 0.001), Vacant Housing Units and Average Household Size (r = −0.823, *p* = 0.000), Vacant Housing Units and Average Money Available per Person (r = 0.898, *p* = 0.000) and Vacant Housing Units and Higher Education Percentage (r = 0.5787, *p* = 0.000) (see Table 2).

Hence, Renter Occupied Housing Units was selected as the independent variable. Mean renter occupancy was higher for the Zika virus present “Yes” group (M = 0.292, SD = 0.100) than Zika present “No” group (M = 0.156, SD = 0.059), t(14) = 3.426, *p* = 0.009. Levene’s Test indicated unequal variances (F = 10.782, *p* = 0.005). The data ware not normally distributed.

The Mann-Whitney Test indicated that mean renter occupancy of the Zika virus present “Yes” group was significantly greater than the Zika virus present “No” group. (U = 6, *p* = 0.008) (see Table 3).

## 4. Discussion

Using the Social Determinants of Health as a framework, we sought to explore the potential existence of a relationship between housing and Zika virus. Our findings indicated a significant correlation existed between renter occupied household units and the presence of Zika virus. This finding is notable as it indicates that renters have a higher chance of contracting Zika virus than non-renters.

Exploring why renters have a higher chance of contracting Zika virus is essential to public health efforts. Specifically, understanding the demographics of renters will enable us to make progress towards prevention. We believe that one of the most significant characteristics of renter demographics to consider is income. According to the Joint Center for Houston of Harvard University, renters are becoming increasingly concentrated at the lowest income levels. By 2010, approximately 70% of renter households had incomes below the national median and more than 40% had incomes in the bottom quartile [11]. Furthermore, the National Multi Family Housing Council reported that 51% of renters had a household income less than $34,999 per year [12].

Income-related constraints result in several notable challenges. Families and individuals with tight budgets may experience financial barriers to moving to a better housing [10]. Because they cannot afford to move, they may be forced to live in rental units with substandard or unhealthy conditions, such as housing without air-conditioning, window screens or proper insulation. Such housing conditions provide the perfect opportunity for airborne vectors, such as *Ae. aegypti* and *Ae. albopictus* (the mosquitoes responsible for the spread of the Zika virus) to enter living units.

In addition to the housing itself, the neighborhoods in which affordable housing is available may experience community-level health-related circumstances that contribute to the spread of the Zika virus and other communicable diseases spread by the *Ae. aegypti* and *Ae. albopictus*, in particular Dengue, Chikungunya and yellow fever. Insufficient sanitation such as inadequate trash collection or illegal dumping can contribute to the spread of the Zika virus by collecting rainwater and providing a breeding ground for mosquitoes. Therefore, the conditions of the neighborhood coupled with the conditions of affordable housing leaves renters vulnerable to conditions that are prime for the spread of Zika and other communicable diseases [6,13,14].

## 5. Limitations

Results of this study are limited due to the small sample size. Future research should examine the demographic and housing situation in other communities reporting cases of the Zika virus.

## Figures and Tables

**Table 1:** Zika virus Count in 16 Zip Code in Cameron County.

**Zip Code**	**78520**	**78521**	**78526**	**78535**	**78550**	**78552**	**78559**	**78566**
Zika Virus Count	12	12	0	0	2	0	2	2
**Zip Code**	**78567**	**78575**	**78578**	**78583**	**78586**	**78592**	**78593**	**78597**
Zika Virus Count	0	0	0	0	2	0	0	0

**Table 2:** Ranks.

	Zika Present	N	Mean Rank	Sum of Ranks
Renter Occupied	0	10	6.10	61.00
	1	6	12.50	75.00
	Total	16		

**Table 3:** Mann-Whitney Test Statistics.

	Renter Occupied
Mann-Whitney U	6.000
Wilcoxon W	61.000
Z	−2.652
Asymp. Sig. (2-tailed)	0.008
Exact Sig. (2-tailed)	0.007 ^a^

^a^ Exact Significance is not corrected for ties.
